# Reviews

**Published:** 1864-08

**Authors:** 


### REVIEWS.

*Medical Diagnosis, with special reference to Practical Medicine; a Guide to the Knowledge and Discrimation of Diseases. By* J. M. DA COSTA, M. D., *Lecturer on Clinical Medicine, and Physician to the Philadelphia Hospital; Fellow to the College of Physicians of Philadelphia; Corresponding Member of the New York Pathological Society, etc., etc., Illustrated with Engravings on wood. Philadelphia:* J. B. LIPPINCOTT & Co., 1864.

This work ls divided into thirteen chapters, in which are considered the symptoms of nearly all diseases recognized by physicians. The first chapter considers the general examination of patients and some of the symptoms of geueral import, such as position of the body, expression of countenance, features of the skin, pulse, tongue, and the general sensations of patients. The second chapter is devoted to the brain, spinal cord, and their nerves, considering the import of *deranged intellection*, delirium, stupor, coma, insomnia; *deranged sensation*—hyperæsthesia, anæsthesia, headache, vertigo, and derangement of special senses; deranged motion—paralysis, tremor, spasms, convulsions; acute affections of which delirium is a prominent symptom; diseases marked by loss of consciousness and voluntary motion—apoplexy, sun-stroke, catalepsy; convulsive diseases—epilepsy, chorea hysteria, tetanus; diseases characterized by gradual impairment of the mental faculties—chronic softening, tumor, general paralysis; diseases characterized by enlargement of the head—chronic hydrocephalus, hypertrophy of the brain; diseases characterized by paroxysmal pain—neuralgia, facial neuralgia, hemicrania, sciatica.

Chapter third is upon diseases of the upper air-passages—larynx and trachea. Chapter fourth on the diseases of the chest, lungs and heart. Chapter fifth, diseases of the mouth, pharynx and œsophagus. Chapter sixth, on diseases of the abdomen, stomach, intestines, peritoneum and liver, with sections on abdominal enlargement and abdominal pulsation. Chapter seven on the urine and on the diseases of the urinary organs. Chapter eighth, dropsy. Ninth, diseases of the blood. Tenth, rheumatism and gout. Eleventh, fevers. Twelfth, diseases of the skin. The thirteenth chapter is devoted to poisons and parasites.

One of the features of this work is the grouping of morbid states, according to their marked symptoms, and a consideration of the different causes of these various symptoms, which is a digression from the usual plan of describing morbid states according to the pathological classification. This is a great improvement upon the plan usually adopted.

The various symptoms of disease are considered with reference to their different causes and the pathological conditions which they indicate. Points of differential diagnosis are made very plain; greatly adding to the practical value of the work.

The illustrations are original, well planned and executed. The chapters upon diseases of the lungs and heart are illustrated in such manner as can not fail to benefit not only the student of medicine, but physicians in active practice will be instructed and greatly assisted by careful perusal. It contains about seven hundred pages, is printed on good paper, with clear type, and makes an elegant volume which should be placed in every medical library, and which will no doubt receive a permanent place in medical literature.

---

*On Rheumatism, Rheumatic Gout and Sciatica, their Pathology, Symptoms and Treatment. By* Henry William Fuller, M. D., *Cantab., Fellow of the Royal College of Physicians, London; Physician to St. George's Hospital, etc., etc. From the last London edition. Philadelphia:* Lindsay & Blakiston, 1864.

It is well to have attention called to the nature, causes, and treatment of a disease which, like rheumatism, is regarded as obscure in its origin, uncertain and variable in its course, and its treatment useless or unsatisfactory. The first chapter in this book is devoted to a consideration of the causes of rheumatism. Objections are urged to the theory that it is produced by the influence of cold or moisture, or both these combined. Argument is made to show that it is due to the presence of morbid matter in the blood; this matter being the product of mal-assimilation or vicious metamorphic action. Lactic acid is regarded as the probable poison. The poison which produces rheumatism is regarded as specific, and as uniform in its action as that which produces small pox or measles, that it is in all cases the same. He says: "But although a specific poison, generated in the system as the result of faulty metamorphic action, is the primary proximate cause of rheumatism, and constitutes the actual materies morbi; yet many agencies may conduce to the formation of the poison, and to its retention in the system, and many circumstances may render the body peculiarly susceptible of its influence. These are the acting and predisposing causes of the disease."

Chapter second is on the rheumatic deathises, and the causes which influence its development. Third; on the seat of rheumatism, and the classification of its different varieties. Fourth, is on acute rheumatism, or rheumatic fever. Fifth is devoted to a consideration of the various remedies which have been employed; and the reasons why treatment has hitherto been contradictory and uncertain. The following remedies are included in the list, and their value and manner of administration described: bleeding, purging, opium, baths, mercury, tartar emetic, cinchona, colchicum, nitrate of potass, lemon-juice, alkalies and their salts. These remedies are approved in some instances, and illustrative cases are cited showing the treatment to be pursued.

Chapters sixth, seventh, eighth and ninth, are very valuable ones, and are devoted to a full consideration of the causes, pathological effects, symptoms, progress, treatment and termination of rheumatic inflammation of the heart.

Chapter tenth is on affections of the brain, inflammation of the lungs and pluræ, and disorganization of the joints, as complications and consequences of acute rheumatism. Rheumatic gout, chronic rheumatism, sciatica and other forms of rheumatic neuralgia, with the treatment to be adopted, are embodied in the three last chapters.

This book is philosophical, well written, eminently practical, widely comprehensive and well worthy the attention of the profession. It treats a class of diseases which physicians should study, and there is no book on this subject, to our knowledge, which so highly commends itself for careful attention and study.

---

*A Manual of the Practice of Medicine. By* THOMAS HAWKES TANNER, M. D., F. L. S., *Member of the Royal College of Physicians; Assistant Physician for the Diseases of Women and Children to King's College Hospital, etc., etc. From the last London edition, enlarged and improved. Philadelphia:* LINDSAY & BLAKISTON, 1864.

This is a clearly written, plain, concise, comprehensive, condensed treatise upon the Practice of Medicine, and though it is unpretending, it is yet one of the most practical and valuable books. The character, symptoms, causes, complications and treatment of diseases are faithfully and quite fully presented, and a wide range of diseases have been introduced, and thus considered in a work modestly denominated a manual.

The student will find it correct and sufficiently comprehensive, while the physician engaged in active practice will often obtain great assistance by its perusal. It is especially adapted to the wants of the practical physician, who has little time to read the larger works; who wants to learn in the shortest possible time the greatest possible amount. The book contains a brief account of nearly every known disease with its appropriate treatment, and an appendix of formulæ, containing proper form of prescribing nearly every known valuable remedy; it contains the greatest conceivable amount of instruction, considering the space occupied.

